# Supplementary material for: A human embryonic limb cell atlas resolved in space and time
Source: Nature. 2023 Dec 6;635(8039):668–78. doi: 10.1038/s41586-023-06806-x (PMC7616500; doi:10.1038/s41586-023-06806-x)
Supplement: Supplementary file 1 — This file contains a table containing cluster label abbreviations, Supplementary Figure 1 (gating strategy for sorting myoblasts), Supplementary Discussion, full legend for Supplementary Video 1 and Supplementary References for Supplementary Table 3. [file 41586_2023_6806_MOESM1_ESM.docx]

**SI Table 1. Cluster label abbreviations**

| **Cluster name** | **Short form** |
| --- | --- |
| **Proximal Mesenchymal Cell** | **ProxMes** |
| **Mesenchymal Cell 1** | **Mes1** |
| **Mesenchymal Cell 2** | **Mes2** |
| **Mesenchymal Cell 3** | **Mes3** |
| **Mesenchymal Cell 4** | **Mes4** |
| **ISL1+Mesenchymal Cell** | **ISL1+Mes** |
| **Transitional Mesenchymal Cell** | **TransMes** |
| **RDH10+ Distal Mesenchymal Cell** | **RDH10+DistalMes** |
| **Distal Mesenchymal Cell** | **DistalMes** |
| **Mesencondensation cell** | **MesCond** |
| **Osteochondral progenetor** | **OCP** |
| **Interzone Cell** | **InterZone** |
| **Chondrogenic Progenitor** | **ChondroProg** |
| **Resting Chondrocyte** | **RestingChon** |
| **Proliferating Chondrocyte** | **ProlifChon** |
| **Prehypertrophic Chondrocyte** | **PrehyperChon** |
| **Hypertrophic Chondrocyte** | **HyperChon** |
| **Perichondrial Cell** | **PeriChon** |
| **Osteoblast** | **OsteoB** |
| **Articular Chondrocyte** | **ArtiChon** |
| **Tendon Progenitor** | **TenoProg** |
| **Tenocyte** | **Teno** |
| **Perimysium Cell** | **Perimysium** |
| **PAX3+ Myogenic Progenitor** | **PAX3+MyoProg** |
| **PAX7+ Myogenic Progenitor** | **PAX7+MyoProg** |
| **Myoblast 1** | **MyoB1** |
| **Myoblast 2** | **MyoB2** |
| **Myocyte 1** | **MyoC1** |
| **Myocyte 2** | **MyoC2** |
| **MYH3+ Myocyte** | **MYH3+MyoC** |
| **MYL3+ Myocyte** | **MYL3+MyoC** |
| **Intermediate Muscular Fibroblast** | **InterMusFibro** |
| **ADH+ Fibroblast** | **ADH+Fibro** |
| **STMN2+ Fibroblast** | **STMN2+Fibro** |
| **MFAP5+ Fibroblast** | **MFAP5+Fibro** |
| **HOXC5+ Dermal Fibroblast Progenitor** | **HOXC5+DermFibroProg** |
| **F10+ Dermal Fibroblast Progenitor** | **F10+DermFibroProg** |
| **Dermal Fibroblast** | **DermFibro** |
| **Smooth Muscle Progenitor** | **SMProg** |
| **Smooth Muscle Cell** | **SMC** |
| **Pericyte** | **Pericyte** |
| **Neural Fibroblast** | **NeuralFibro** |
| **Neuronal Cell** | **Neuronal** |
| **Schwann Progenitor** | **SchwannProg** |
| **Schwann Cell** | **Schwann** |
| **Synaptic Schwann** | **SynapSchwann** |
| **Melanocyte** | **Melano** |
| **Periderm Cell** | **Periderm** |
| **Basal Cell** | **Basal** |
| **Apical Ectodermal Ridge Basal Cell** | **AER-Basal** |
| **Arterial Endothelial Cell** | **ArterialEndo** |
| **Venous Endothelial Cell** | **VenousEndo** |
| **Lymphatic Endothelial Cell** | **LymphEndo** |
| **Lymphoid-primed multipotent progenitor** | **LMPP/ELP** |
| **Common Myeloid Progenitor/Granulocyte-monocyte Progenitor** | **CMP/GMP** |
| **Natural Killer Cell** | **NK** |
| **B Cell** | **B** |
| **Myelocyte** | **Myelocyte** |
| **Dendritic Cell 2** | **DC2** |
| **Monocyte** | **Monocyte** |
| **Macrophage** | **Macro** |
| **Mast Cell** | **Mast** |
| **Megakaryocyte** | **Megakaryo** |
| **Definite Erythrocyte** | **DefErythro** |
| **Definite Reticulocyte** | **DefReticulo** |
| **Primitive Erythrocyte 1** | **PrimErythro1** |
| **Primitive Erythrocyte 2** | **PrimErythro2** |

**SI Figure 1.** **Gating strategy for sorting myoblasts**

**
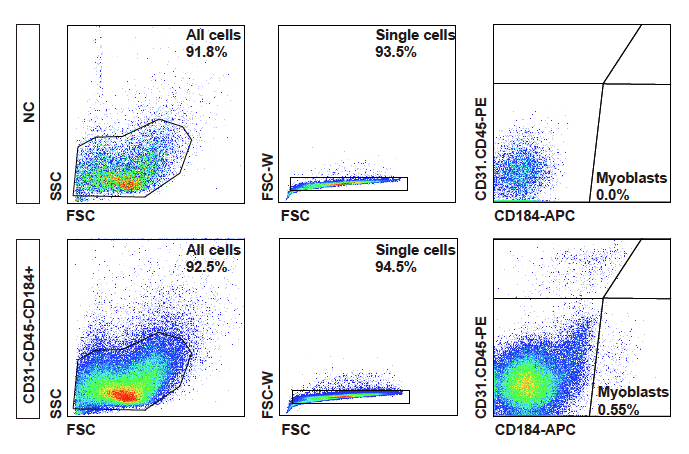
**

**Supplementary Discussion**. **Limitations of this study**

One limitation in our study is the lack of samples from the earliest stages of limb bud development. Although we were able to dissect and process limbs from PCW5.1 onwards, the logistical limitations of working with human embryonic tissue precluded analysis of the nascent limb bud and the earliest patterning and maturation events, such as the first wave of *HOX* gene expression and early *FGF8* expression in the distal ectoderm. Furthermore, whilst we were able to investigate *FGFR2* expression patterns in the limb, the assays used are unable at present to distinguish between its IIIb (highest *FGF10* affinity) and IIIc (highest *FGF8* affinity) isoforms.

Whilst the combination of single cell and spatial transcriptomics is an established method for tissue atlasing, we recognise the challenges of combining these technologies. For example, our single-cell data captured large numbers of chondrocytes but only n=14 cells expressing collagen-X, which is disproportionately smaller than expected based on Visium slides. One possible explanation for this is that permeabilisation and RNA capture with Visium was superior to the single cell tissue dissociation and droplet-based capture method for profiling matrix-rich tissues such as cartilage. Another important factor to consider is the breadth of cell capture with each technique. For scRNAseq, a typical cell suspension contains hundreds of thousands of cells, only a fraction of which are sequenced, meaning rarer populations are unlikely to be profiled. By contrast, ST captures RNA from all cells within each voxel across an entire tissue section, thus rare cell populations should still contribute to the overall RNA signal. These factors may also explain the low numbers of SCX^+^/SOX9^+^ enthesis progenitor cells captured in our study. We expect these technical considerations to feed forward into future atlasing endeavours involving matrix-rich tissue, or where in-depth transcriptomic profiling of rare cell types is desired.

Although spatial transcriptomics gives valuable anatomical context to sequenced single cells, it is limited in its utility due to its 50μm resolution (up to 25 cells per voxel in the limb), as well as significant dead space between voxels that is not sequenced. This can result in uncertain patterns of location calling or gene expression (such as macrophages and endothelium in the limb, Extended Data Fig. 6g, h), requiring further validation. Thus a true understanding of tissue architecture at the single cell, whole transcriptome level remains elusive. Furthermore, such a large sampling area makes fine-grain analyses of gene expression based on histology or anatomy challenging. In this study, we attempted to identify genes that vary between individual digits, but found very few. This is likely due to a lack of statistical power, with each digit only occupying 10-16 voxels. Similarly, our comparative analyses of digit and interdigit spaces was limited by any voxels that exhibited even a small degree of overlap (and hence transcript mixing) between the two tissues.

**Legend for supplementary video 1**

light sheet fluorescence microscopy of marker genes for mesenchyme populations in the developing autopod.

Clip1: Immunostaining of MSX1, IRX1 and SOX9 in the PCW5 human fetal foot plate.

Clip2: Immunostaining of *MSX1*, *IRX1* and *SOX9* in the PCW6 human fetal foot plate.

Clip3: Immunostaining of *MSX1*, *IRX1* and *SOX9* in the PCW6 human fetal hand plate

**Supplementary references for Extended Data Table 3**

S1. [Eiken, M., Prag, J., Petersen, K. E. & Kaufmann, H. J. A new familial skeletal dysplasia with severely retarded ossification and abnormal modeling of bones especially of the epiphyses, the hands, and feet. *Eur. J. Pediatr.* **141**, (1984).](http://paperpile.com/b/aL5t0O/Jo4MV)

S2. [Fan, Y. *et al.* Parathyroid hormone 1 receptor is essential to induce FGF23 production and maintain systemic mineral ion homeostasis. *The FASEB Journal* **30**, 428 (2016).](http://paperpile.com/b/aL5t0O/HGcvl)

S3. [Yi, S. E., Daluiski, A., Pederson, R., Rosen, V. & Lyons, K. M. The type I BMP receptor BMPRIB is required for chondrogenesis in the mouse limb. *Development* vol. 127 621–630 Preprint at https://doi.org/](http://paperpile.com/b/aL5t0O/OpHVQ)[10.1242/dev.127.3.621](http://dx.doi.org/10.1242/dev.127.3.621) [(2000).](http://paperpile.com/b/aL5t0O/OpHVQ)

S4. [Stafford, D. A., Brunet, L. J., Khokha, M. K., Economides, A. N. & Harland, R. M. Cooperative activity of noggin and gremlin 1 in axial skeleton development. *Development* **138**, 1005–1014 (2011).](http://paperpile.com/b/aL5t0O/xirYH)

S5. [Brunet, L. J., McMahon, J. A., McMahon, A. P. & Harland, R. M. Noggin, Cartilage Morphogenesis, and Joint Formation in the Mammalian Skeleton. *Science* vol. 280 1455–1457 Preprint at https://doi.org/](http://paperpile.com/b/aL5t0O/6rU6N)[10.1126/science.280.5368.1455](http://dx.doi.org/10.1126/science.280.5368.1455) [(1998).](http://paperpile.com/b/aL5t0O/6rU6N)

S6. [Melkoniemi, M. *et al.* Autosomal recessive disorder otospondylomegaepiphyseal dysplasia is associated with loss-of-function mutations in the COL11A2 gene. *Am. J. Hum. Genet.* **66**, 368–377 (2000).](http://paperpile.com/b/aL5t0O/aYcxr)

S7. [Li, S. W. *et al.* Targeted disruption of Col11a2 produces a mild cartilage phenotype in transgenic mice: comparison with the human disorder otospondylomegaepiphyseal dysplasia (OSMED). *Dev. Dyn.* **222**, 141–152 (2001).](http://paperpile.com/b/aL5t0O/uinIG)

S8. [Bi, W. *et al.* Haploinsufficiency of Sox9 results in defective cartilage primordia and premature skeletal mineralization. *Proc. Natl. Acad. Sci. U. S. A.* **98**, 6698–6703 (2001).](http://paperpile.com/b/aL5t0O/vWWTX)

S9. [Shazeeb, M. S. *et al.* Skeletal Characterization of the Fgfr3 Mouse Model of Achondroplasia Using Micro-CT and MRI Volumetric Imaging. *Scientific Reports* vol. 8 Preprint at https://doi.org/](http://paperpile.com/b/aL5t0O/xPQBX)[10.1038/s41598-017-18801-0](http://dx.doi.org/10.1038/s41598-017-18801-0) [(2018).](http://paperpile.com/b/aL5t0O/xPQBX)

S10. [Carey, J. C., Cassidy, S. B., Battaglia, A. & Viskochil, D. *Cassidy and Allanson’s Management of Genetic Syndromes*. (John Wiley & Sons, 2021).](http://paperpile.com/b/aL5t0O/v1if2)

S11. [Sowińska-Seidler, A., Socha, M. & Jamsheer, A. Split-hand/foot malformation - molecular cause and implications in genetic counseling. *J. Appl. Genet.* **55**, 105–115 (2014).](http://paperpile.com/b/aL5t0O/cRB8X)

S12. [Merlo, G. R. *et al.* Mouse model of split hand/foot malformation type I. *Genesis* **33**, 97–101 (2002).](http://paperpile.com/b/aL5t0O/kZGIo)

S13. [Celli, J., van Bokhoven, H. & Brunner, H. G. Feingold syndrome: Clinical review and genetic mapping. *American Journal of Medical Genetics* vol. 122A 294–300 Preprint at https://doi.org/](http://paperpile.com/b/aL5t0O/999Nj)[10.1002/ajmg.a.20471](http://dx.doi.org/10.1002/ajmg.a.20471) [(2003).](http://paperpile.com/b/aL5t0O/999Nj)

S14. [Sawai, S. *et al.* Defects of embryonic organogenesis resulting from targeted disruption of the N-myc gene in the mouse. *Development* **117**, 1445–1455 (1993).](http://paperpile.com/b/aL5t0O/DuquZ)

S15. [Selever, J., Liu, W., Lu, M.-F., Behringer, R. R. & Martin, J. F. Bmp4 in limb bud mesoderm regulates digit pattern by controlling AER development. *Developmental Biology* vol. 276 268–279 Preprint at https://doi.org/](http://paperpile.com/b/aL5t0O/YXRhS)[10.1016/j.ydbio.2004.08.024](http://dx.doi.org/10.1016/j.ydbio.2004.08.024) [(2004).](http://paperpile.com/b/aL5t0O/YXRhS)

S16. [Gripp, K. W., Zackai, E. H. & Stolle, C. A. Mutations in the human TWIST gene. *Hum. Mutat.* **15**, 479 (2000).](http://paperpile.com/b/aL5t0O/tIdKc)

S17. [Bialek, P. *et al.* A Twist Code Determines the Onset of Osteoblast Differentiation. *Developmental Cell* vol. 6 423–435 Preprint at https://doi.org/](http://paperpile.com/b/aL5t0O/UfS1Q)[10.1016/s1534-5807(04)00058-9](http://dx.doi.org/10.1016/s1534-5807(04)00058-9) [(2004).](http://paperpile.com/b/aL5t0O/UfS1Q)
