# Supplementary material for: A human embryonic limb cell atlas resolved in space and time
Source: Nature. Author manuscript; Available in PMC 2024 Nov 21. (PMC7616500; doi:10.1038/s41586-023-06806-x)
Supplement: Supplementary file 1 [file EMS193945-supplement-Supplementary_Information.pdf]

nature portfolio

Corresponding author(s): Hongbo Zhang and Sarah Teichmann

Last updated by author(s): Sep 4, 2023

Reporting Summary

Nature Portfolio wishes to improve the reproducibility of the work that we publish. This form provides structure for consistency and transparency in reporting. For further information on Nature Portfolio policies, see our Editorial Policies and the Editorial Policy Checklist.

Statistics

For all statistical analyses, confirm that the following items are present in the figure legend, table legend, main text, or Methods section.

n/a	Confirmed	
☐	☒	The exact sample size (*n*) for each experimental group/condition, given as a discrete number and unit of measurement
☐	☒	A statement on whether measurements were taken from distinct samples or whether the same sample was measured repeatedly
☐	☒	The statistical test(s) used AND whether they are one- or two-sided<br>*Only common tests should be described solely by name; describe more complex techniques in the Methods section.*
☐	☒	A description of all covariates tested
☐	☒	A description of any assumptions or corrections, such as tests of normality and adjustment for multiple comparisons
☐	☒	A full description of the statistical parameters including central tendency (e.g. means) or other basic estimates (e.g. regression coefficient) AND variation (e.g. standard deviation) or associated estimates of uncertainty (e.g. confidence intervals)
☐	☒	For null hypothesis testing, the test statistic (e.g. *F*, *t*, *r*) with confidence intervals, effect sizes, degrees of freedom and *P* value noted<br>*Give P values as exact values whenever suitable.*
☒	☐	For Bayesian analysis, information on the choice of priors and Markov chain Monte Carlo settings
☐	☒	For hierarchical and complex designs, identification of the appropriate level for tests and full reporting of outcomes
☒	☐	Estimates of effect sizes (e.g. Cohen's *d*, Pearson's *r*), indicating how they were calculated

Our web collection on statistics for biologists contains articles on many of the points above.

Software and code

Policy information about availability of computer code

Data collection	Software used for data alignment and mapping include: STAR (v2.5.1b52) , 10X Space Ranger software (v.1.1.0)
Data analysis	Single cell data analysis was mostly performed using Python (v3.7.4) and scanpy (v1.8.2).<br>scRNA-seq data were aligned by Cellranger (v3.0.2) with hg38 (GRCh38-3.0.0) and mm10 (mm10-2020-A).<br>Visium data were aligned using Space Ranger (v1.1.0)<br>Doublets were removed using Scrublet (v0.2.1).<br>Batch correction was performed using bbknn (v1.5.1).<br>RNA velocity analysis was done by scVelo(v0.24)<br>data integration of human and mouse was performed using MultiMAP (v1.0).<br>Cell-cell communication analysis was performed using CellPhoneDB (v2.1.4).<br>Enrichment analysis of transcription factors was performed using pySCENIC (v0.10.3).<br>Alignment and merging of multiple visium sections was performed using Fiji.<br>Deconvolution of human Visium data was performed using cell2location (v0.1).<br>RNA Velocity analysis was performed using scVelo (v0.24).<br>Colocalisation analysis was done using ilastik (v1.3)<br>Light Sheet Fluorescence Microscopy was supported by Imspector Pro (v7.5.3) and Imaris (v10.0)<br>Flow cytometry analysis was done using FlowJo (v10)<br>MSC knock-down data were plotted using Prism (v9)

For manuscripts utilizing custom algorithms or software that are central to the research but not yet described in published literature, software must be made available to editors and reviewers. We strongly encourage code deposition in a community repository (e.g. GitHub). See the Nature Portfolio guidelines for submitting code & software for further information.

Data

Policy information about availability of data

All manuscripts must include a data availability statement. This statement should provide the following information, where applicable:

- Accession codes, unique identifiers, or web links for publicly available datasets
- A description of any restrictions on data availability
- For clinical datasets or third party data, please ensure that the statement adheres to our policy

All of our newly generated raw data are publicly available on ArrayExpress (mouse scRNA-seq, E-MTAB-10514; human Visium, E-MTAB-10367; human scRNA-seq, E-MTAB-8813). Previously published raw data can be found from ENCODE portal (ENCSR713GIS, https://www.encodeproject.org/publication-data/ENCSR713GIS/) and GEO (GSE137335 https://www.ncbi.nlm.nih.gov/geo/query/acc.cgi?acc=GSE137335 and GSE142425 https://www.ncbi.nlm.nih.gov/geo/query/acc.cgi?acc=GSE142425). Processed data and be downloaded and visualisedvisualized at our data portal (https://limb-dev.cellgeni.sanger.ac.uk/). All the source data for figures can be found in supplementary Excel files.

Research involving human participants, their data, or biological material

Policy information about studies with human participants or human data. See also policy information about sex, gender (identity/presentation), and sexual orientation and race, ethnicity and racism.

Reporting on sex and gender	This study focuses on the limb development of human embryo, and we included data from medical aborted female and male embryos. From the stage of PCW5-9 we have analysed, we do not observe sex and gender difference between females and males.
Reporting on race, ethnicity, or other socially relevant groupings	*Please specify the socially constructed or socially relevant categorization variable(s) used in your manuscript and explain why they were used. Please note that such variables should not be used as proxies for other socially constructed/relevant variables (for example, race or ethnicity should not be used as a proxy for socioeconomic status).*<br>*Provide clear definitions of the relevant terms used, how they were provided (by the participants/respondents, the researchers, or third parties), and the method(s) used to classify people into the different categories (e.g. self-report, census or administrative data, social media data, etc.)*<br>*Please provide details about how you controlled for confounding variables in your analyses.*
Population characteristics	We used first trimister embyros (age, post conception weeks 5-9) from voluntary medical abortion. Tissue samples used for human scRNA-seq/Visum and validation experiments were obtained from donors of British and Chinese, respectively. No developmental abnormalities were visible or known in any of the embryos collected.
Recruitment	Medical aborted embryos were collected with the agreement of the pregnant female. The termination time point were decided by the female and doctor.
Ethics oversight	First trimester human embryonic tissue was collected from elective termination of pregnancy procedures at Addenbrookes Hospital, Cambridge, UK under full ethical approval (REC-96/085; for scRNA-seq and Visium), at Guangzhou Women and Children's Medical Center, China under approval of the Research Ethics Committee of Sun Yat-sen University (ZSSOM-2019-075) and Guangzhou Women and Children's Medical Centre (2022-050A01, for In-situ hybridisation and immunohistochemistry). Experiments were also followed the 2021 International Society for Stem Cell Research (ISSCR) guidelines in working on human embryos. Informed written consent was obtained from all donors before abortion and tissue collection. No developmental abnormalities were visible or known in any of the embryos collected. All human data generated from China was registered at China National Center for Bioinformation (PRJCA012474) and has been approved by the Chinese Ministry of Science and Technology for the Review and the Approval of Human Genetic Resources (2023BAT0445). For light-sheet fluorescence microscopy, tissues were obtained through INSERM’s HuDeCA Biobank and made available in accordance with the French bylaw. Permission to use human tissues was obtained from the French agency for biomedical research (Agence de la Biomédecine, Saint-Denis La Plaine, France; N° PFS19-012) and INSERM Ethics Committee (IRB00003888). Written, informed consent was given for tissue collection by all patients. Embryonic age (post conception weeks, PCW) was estimated using the independent measurement of the crown rump length (CRL), using the formula PCW (days) = 0.9022 × CRL (mm) + 27.372.

Note that full information on the approval of the study protocol must also be provided in the manuscript.

Field-specific reporting

Please select the one below that is the best fit for your research. If you are not sure, read the appropriate sections before making your selection.

☒ Life sciences ☐ Behavioural & social sciences ☐ Ecological, evolutionary & environmental sciences

For a reference copy of the document with all sections, see nature.com/documents/nr-reporting-summary-flat.pdf

Life sciences study design

All studies must disclose on these points even when the disclosure is negative.

Sample size	For human scRNA seq, n = 1 at PCW5.1; n = 2 at PCW5.4; n = 6 at PCW5.6; n = 6 at PCW6.1; n = 1 at PCW6.5; n = 2 at PCW7.2; n = 1 at PCW8.0; n = 2 at PCW8.4; n = 2 at PCW9.0; n = 2 at PCW9.3;.<br>For human Visum, n = 2 at PCW5.6; n = 3 at PCW6.2; n = 2 at PCW7.0; n = 4 at PCW8.1;<br>For mouse scRNA seq, n = 5 at E12.5; n = 5 at E13.5; n = 2 at E16.5.<br>For experimental validation:<br>RNA In situ hybridization: n = 2-4 for each staining at indicated stage mentioned in the manuscript;<br>Light Sheet Fluorescence Microscopy for MSX1 IRX1 SOX9 staining: n = 2;<br>RUNX2, THBS2, COL2A1 immunofluorescence staining: n = 3;<br>PITX1, PAX3 immunofluorescence staining: hindlimb, n = 4; forelimb, n = 2;<br>ALDH1A3, MYH3 immunofluorescence staining: n = 2;<br>PI16, FGF19, NEFH Immunohistochemistry staining: n = 4;<br>Myoblast isolation and culture: n = 2.<br>GCG, MYH3, KERA staining: n = 2<br>Sample size depends on availability of human tissues. We try to include at least two replicates when available. No sample size calculation was performed.
Data exclusions	We excluded cells based on the QC thresholds summarized in Methods section. We also removed cell doublets.
Replication	We used 1-2 biological and 1-2 technical replicates for human scRNA-seq and Visum;<br>We used 1-6 biological and 1-2 technical replicates for mouse scRNA-seq.<br>We used 2-4 biological and 1-3 technical replicates for experimental validations.<br>All attempts at replication were successful.
Randomization	Intentional randomization was not performed. Samples were allocated based on their ages.
Blinding	All human specimens were de-identified before analyses. However, selected attributes such as (developmental stage and dissected region) were available to all investigators. Blinding was not performed during tissue sample collection, analysis of scRNA-seq and Visium, as well as experimental validations, although our initial computational processing used unbiased approaches for all the sequencing samples. A majority of the downstream analyses did not adopt blinding as key sample attributes were needed for accurate cell cluster annotation and downstream analyses to create the atlas.

Reporting for specific materials, systems and methods

We require information from authors about some types of materials, experimental systems and methods used in many studies. Here, indicate whether each material, system or method listed is relevant to your study. If you are not sure if a list item applies to your research, read the appropriate section before selecting a response.

Materials & experimental systems

n/a	Involved in the study	
☐	☒	Antibodies
☒	☐	Eukaryotic cell lines
☒	☐	Palaeontology and archaeology
☐	☒	Animals and other organisms
☒	☐	Clinical data
☒	☐	Dual use research of concern
☒	☐	Plants

Methods

n/a	Involved in the study	
☒	☐	ChIP-seq
☐	☒	Flow cytometry
☒	☐	MRI-based neuroimaging

Antibodies

Antibodies used	1. Anti-RUNX2 Antibody (C-12) (1:50, Santa Cruz, sc-390715)<br>https://www.scbt.com/p/runx2-antibody-c-12<br>2. Anti-THBS2 (Thrombospondin 2) Polyclonal Antibody (1:100, Invitrogen, PA5-76418)<br>https://www.thermofisher.cn/cn/zh/antibody/product/Thrombospondin-2-Antibody-Polyclonal/PA5-76418<br>3. Anti-COL2A1 (M2139) antibody (1:200, Santa Cruz, sc-52658)<br>https://www.scbt.com/p/col2a1-antibody-m2139/<br>4. Anti-PITX1/BFT antibody (1:30, Abcam, ab244308)<br>https://www.abcam.com/pitx1bft-antibody-ab244308.html<br>5. Anti-PAX3 antibody (1:1, DSHB, AB_528426 supernatant)<br>https://dshb.biology.uiowa.edu/Pax3<br>6. Anti-ALDH1A3 antibody (1:50, Proteintech, 25167-1-AP)<br>https://www.ptglab.com/Products/ALDH1A3-Antibody-25167-1-AP.htm<br>7. Anti-MYH3 (F1.652) antibody (1:3, DSHB, AB_528358 supernatant)<br>8. Anti-PI16 antibody (1:500, Sigma-Aldrich, HPA043763)

https://www.sigmaaldrich.com/catalog/product/sigma/hpa043763
9. Anti-FGF19 antibody (1:500, Affinity, DF2651)
http://www.affbiotech.com/goods-6732-DF2651-FGF19_Antibody.html
10. Anti-NEFH (Neurofilament-H) (RMdO 20)antibody (1:1000, Cell Signaling, 2836)
https://www.cellsignal.com/products/primary-antibodies/neurofilament-h-rmdo-20-mouse-mab/2836
11. Alexa Flour 488 goat anti-mouse IgG1 (1:400, Invitrogen, A-21121)
https://www.thermofisher.cn/cn/zh/antibody/product/Goat-anti-Mouse-IgG1-Cross-Adsorbed-Secondary-Antibody-Polyclonal/A-21121
12. Alexa Flour 647 goat anti-mouse IgG2b (1:400, Invitrogen, A-21242)
https://www.thermofisher.cn/cn/zh/antibody/product/Goat-anti-Mouse-IgG2b-Cross-Adsorbed-Secondary-Antibody-Polyclonal/A-21242
13. Alexa Flour 488 goat anti-mouse IgG (H+L) (1:400, Invitrogen, A-11029)
https://www.citeab.com/antibodies/2401117-a-11029-goat-anti-mouse-igg-h-l-highly-cross-adsor
14. Alexa Flour 546 goat anti-rabbit IgG (H+L) (1:400, Invitrogen, A-11035)
https://www.thermofisher.cn/cn/zh/antibody/product/Goat-anti-Rabbit-IgG-H-L-Highly-Cross-Adsorbed-Secondary-Antibody-Polyclonal/A-11035
15. Streptavidin-Peroxidase broad spectrum Immunohistochemical staining kit (Bioss, SP-0022)
http://www.bioss.com.cn/prolook_03.asp?id=AF08169606008548&pro37=9
16. IRX1 (1:200, Sigma-Aldrich, HPA043160)
https://www.sigmaaldrich.com/US/en/product/sigma/hpa043160
17. MSX1 (1:500, R&D Systems, AF5045)
https://www.rndsystems.com/cn/products/human-mouse-msx1-antibody_af5045
18. LHX2 (1:1000, Abcam, ab184337)
https://securedrtest.abcam.com/products/primary-antibodies/lhx2lh2-antibody-epr20449-ab184337.html
19. SOX9 (1:500, Abcam, ab196184)
https://securedrtest.abcam.com/products/primary-antibodies/alexa-fluor-647-sox9-antibody-epr14335-ab196184.html
20. MAFB (1:500, Abcam, ab223744)
https://securedrtest.abcam.com/products/primary-antibodies/mafb-antibody-ab223744.html
21. Donkey Anti-Rabbit IgG H&L (1:800, Alexa Fluor® 555) (Abcam, ab150062)
https://securedrtest.abcam.com/products/secondary-antibodies/donkey-rabbit-igg-hl-alexa-fluor-555-preadsorbed-ab150062.html
22. Donkey Anti-Goat IgG H&L (Alexa Fluor® 790) (1:300, Abcam, ab175745)
https://securedrtest.abcam.com/products/secondary-antibodies/donkey-goat-igg-hl-alexa-fluor-750-preadsorbed-ab175745.html
23. anti-KERA antibody (1:1000, Sigma-Aldrich, HPA039321)
https://www.sigmaaldrich.cn/CN/en/product/sigma/hpa039321
24. CD31 (PECAM-1) Monoclonal Antibody (WM-59 (WM59)), PE (5 µL/Test, eBioscience, 12-0319-41)
https://www.thermofisher.cn/cn/zh/antibody/product/CD31-PECAM-1-Antibody-clone-WM-59-WM59-Monoclonal/12-0319-41
25. CD45 Monoclonal Antibody (HI30), PE (5 µL/Test, eBioscience, 12-0459-41)
https://www.thermofisher.cn/cn/zh/antibody/product/CD45-Antibody-clone-HI30-Monoclonal/12-0459-41
26. CD184 (CXCR4) Monoclonal Antibody (12G5), APC (5 µL/Test, eBioscience, 17-9999-41)
https://www.thermofisher.cn/cn/zh/antibody/product/CD184-CXCR4-Antibody-clone-12G5-Monoclonal/17-9999-41

Validation

For all the above antibodies used in the manuscript, they relevant applications (i.e. FACS or Immunohistochemistry staining) were validated by the manufactures and indicated on the individual website.

Animals and other research organisms

Policy information about studies involving animals; ARRIVE guidelines recommended for reporting animal research, and Sex and Gender in Research

Laboratory animals

C57BL/6N wild type embryos; All animal procedures were performed according to protocols approved by the Institutional Animal Care and Use Committee at the California Institute of Technology. Animals were housed in an AAALAC accredited facility in accordance with the Guide for the Care and Use of Laboratory Animals. Animal rooms were maintained on a 14:10 h light:dark cycle with an hour-long dawn/dusk period with humidity ranging from 30% to 70% and temperatures ranging from 71 to 75 °F.Age: n = 5 at E12.5; n = 5 at E13.5; n = 2 at E16.5

Wild animals

No wild animals were used in this study

Reporting on sex

Sex information was not recorded at the time of collection due to sample processing time requirements.

Field-collected samples

No field collected samples were used in the study

Ethics oversight

approved by The Institutional Animal Care and Use Committee at the California Institute of Technology

Note that full information on the approval of the study protocol must also be provided in the manuscript.

Flow Cytometry

Plots

Confirm that:

☒ The axis labels state the marker and fluorochrome used (e.g. CD4-FITC).

☒ The axis scales are clearly visible. Include numbers along axes only for bottom left plot of group (a 'group' is an analysis of identical markers).

☒ All plots are contour plots with outliers or pseudocolor plots.

☒ A numerical value for number of cells or percentage (with statistics) is provided.

Methodology

Sample preparation	The thighs from human embryos were processed as described (Lapan AD, et al. Methods Mol Biol. 2012), except that the dissociated cells were not treated with erythrocyte lysis solution, and were incubated with anti-human CD31 (eBioscience, 12-0319-41), CD45 (eBioscience, 12-0459-41) and CD184 (eBioscience, 17-9999-41) antibodies for cell sorting. Fluorescent activated cell sorting (FACS, BD, influx) sorted CD31-CD45-CD184+ cells were cultured in complete growth medium DMEM supplemented with 20% FCS and 1% penicillin/streptomycin (Gibco, 15140122).
Instrument	BD Influx Cell Sorter
Software	Exported raw file was opened with FlowJo (version 10) to analyze cell populations.
Cell population abundance	The number of CD31-CD45- cells accounts for approximately 97.24% of embryonic thigh dissociated cells, and the abundance of CD184+ cells is about 0.56%. The percentage for CD31-CD45- CD184+ cells is about 0.54%.
Gating strategy	Gating for CD31-CD45- CD184+ to isolate myoblasts: 1) gate on FSC vs SSC to exclude cell debris or small particles and include all cell populations.; 2) gate on PE channel to sort CD31-CD45- cells; 2) gate on APC channel to sort CD31-CD45- CD184+ cells as primary myoblasts.

☒ Tick this box to confirm that a figure exemplifying the gating strategy is provided in the Supplementary Information.
